# Recent Advances in Elucidating the Mechanism of the NADPH–Cytochrome P450 Reductase-Mediated Electron Transfer Cycle: Experimental and Computational Perspectives

**DOI:** 10.3390/molecules30183733

**Published:** 2025-09-13

**Authors:** Songyan Xia, Hajime Hirao

**Affiliations:** Warshel Institute for Computational Biology, School of Medicine, The Chinese University of Hong Kong, Shenzhen 518172, China; songyanxia@link.cuhk.edu.cn

**Keywords:** NADPH–cytochrome P450 reductase (CPR), electron transfer mechanism, conformational dynamics, protein–protein interaction, nicotinamide adenine dinucleotide phosphate (NADPH), flavin adenine dinucleotide (FAD), flavin mononucleotide (FMN)

## Abstract

NADPH–cytochrome P450 reductase (CPR) is an essential redox partner for a wide range of metal-containing proteins, mediating the stepwise transfer of two electrons from nicotinamide adenine dinucleotide phosphate (NADPH) to the redox centers of its partner proteins. This Perspective summarizes recent advances in understanding the mechanisms underlying the CPR-mediated electron transfer (ET) cycle. Emphasis is placed on human and other mammalian CPRs, which provide critical insights into human biology and drug metabolism. Recent experimental and computational approaches that have deepened our mechanistic understanding of CPR function are highlighted. Selected studies are reviewed to illustrate progress in elucidating the interflavin ET within CPR, the interplay between its redox states and structural dynamics, and its protein–protein interactions with redox partners, along with the associated ET pathways. Finally, the remaining challenges and future research directions are outlined.

## 1. Introduction

**Figure 1:**
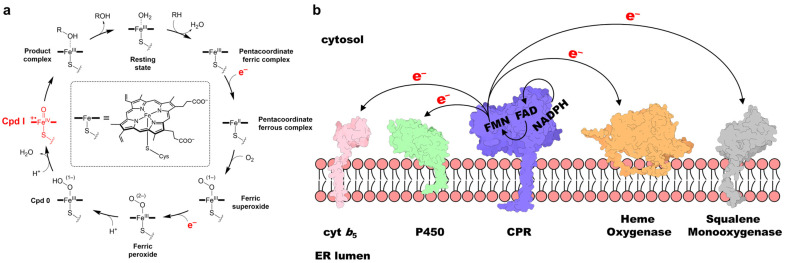
(**a**) Schematic illustration of the P450 catalytic cycle, with two supplied electrons and Cpd I highlighted in red. (**b**) Schematic representation of CPR and its redox partner proteins embedded in the ER membrane. Note that, although shown as spatially separated for illustrative purposes, the actual ET processes occur through protein–protein interactions.

Reduction of the iron in the heme prosthetic group is a critical step in the catalytic function of cytochrome P450 enzymes (P450s) across a diverse range of organisms [1,2,3]. In human P450s, two electrons are transferred from the two-electron donor nicotinamide adenine dinucleotide phosphate (NADPH) to the heme during each catalytic cycle, enabling the formation of the highly reactive intermediate Compound I (Cpd I) and facilitating physiologically and pharmacologically significant oxidative transformations (Figure 1a) of substrates, including endogenous compounds and drugs [2,4,5]. Class II P450s, which are typically found in eukaryotic organisms and localized to the endoplasmic reticulum (ER) membrane (Figure 1b), depend on NADPH–cytochrome P450 reductase (CPR) to mediate electron transfer (ET) from NADPH [6]. CPR also mediates ET to other one-electron acceptor proteins, such as heme oxygenase (HO), cytochrome *b*_5_ (cyt *b*_5_), and squalene mono-oxygenase (Figure 1b) [7,8,9,10,11]. In addition, CPR contributes to the activation of several clinically approved anticancer drugs [12,13] and has emerged as a potential therapeutic target in the regulation of ferroptosis [14,15]. Because of the central importance of these ET processes, extensive research has focused on elucidating CPR’s structure, function, and role in human disease since its initial discovery and characterization in the 1950s [7,16,17,18,19,20].

**Figure 2:**
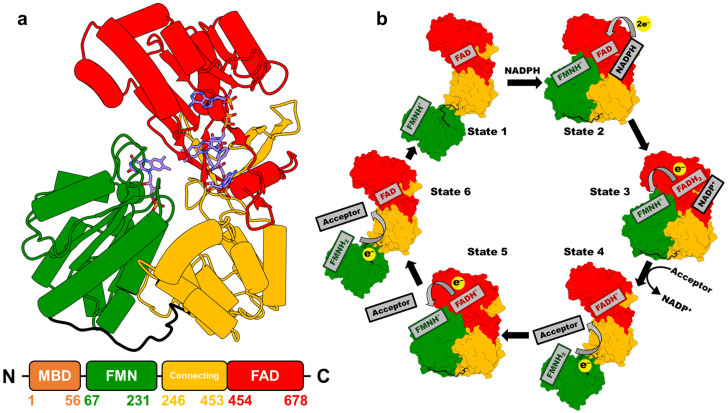
(**a**) Crystal structure of NADP^+^-bound rat CPR in the closed conformation (PDB 4YAL, [11]) [23]. Different domains are distinguished by color: FAD-binding domain (red), connecting domain (yellow), and FMN-binding domain (green). Carbon atoms in FAD, FMN, and NADP^+^ are shown in blue. The lower panel depicts the primary structure of CPR, with the amino acid range indicated for each domain. (**b**) Schematic diagram illustrating the proposed conformational changes and cofactor redox states at different stages of the CPR-mediated ET cycle [21].

Mammalian CPR is a ubiquitous ~78 kDa membrane-bound protein that contains the cofactors flavin adenine dinucleotide (FAD) and flavin mononucleotide (FMN), which are bound to distinct domains (Figure 2a) and serve as essential relay centers for transferring electrons from NADPH to redox partners such as P450s. Initially, two electrons are transferred from NADPH to FAD as a hydride (states **1** to **2** in Figure 2b). These electrons are then passed sequentially from FAD to FMN, generating the FMN hydroquinone intermediate (FMNH_2_ in Figure 2b). The reduced FMN subsequently donates the electrons one at a time to redox partner proteins [21]. This ET cascade activates the partner proteins, enabling them to carry out their biochemical functions [1,7]. 

CPR displays a highly conserved structure and amino acid sequence across biological kingdoms. It is composed of three distinct domains, preceded by an N-terminal membrane anchor [7,22]. The first domain, the FMN-binding domain, is approximately 160 amino acids in length and binds the FMN cofactor (Figure 2a). Adjacent to it is the connecting domain, comprising roughly 220 amino acids, which functions as a structural bridge between the FMN-binding domain and the C-terminal FAD-binding domain. The latter accommodates both the FAD and NADPH cofactors [23].

The CPR-mediated ET process is tightly coupled to large-scale conformational transitions between a compact closed state and an extended open state (Figure 2b) [24]. The closed conformation promotes efficient interflavin ET from FAD to FMN, whereas the open conformation facilitates effective ET from FMN to its redox partners [25,26,27,28]. The mechanistic complexity arises from the interplay of multiple factors, including conformational changes, the redox transitions of the FAD and FMN cofactors, the spatial distribution of local electric fields, and the influence of the lipid membrane environment, making detailed mechanistic elucidation particularly challenging [28,29,30,31,32,33]. Furthermore, the structural diversity and heterogeneity of CPR redox partners further complicate efforts to fully define the regulatory principles governing ET within the CPR-dependent system. Continued research is therefore essential to elucidate the mechanisms underlying CPR function.

In this Perspective, we provide an overview of recent advances and outline future directions in the study of the CPR-mediated ET cycle, with particular focus placed on human and other mammalian CYPs, although the coverage may not be entirely comprehensive. Plant and bacterial CPRs, although sharing overall three-dimensional (3D) structural similarity with mammalian CPR, exhibit relatively low sequence identity and are therefore not within the scope of this Perspective [34,35]. We begin by describing recent experimental and computational techniques that have greatly enhanced the resolution and accuracy of probing CPR’s structural and dynamic properties. These methodological advances have enabled more detailed characterization of CPR’s conformational states, redox transitions, and interactions with redox partner proteins. We then highlight key studies that reveal emerging trends in CPR research, with particular emphasis on the coupling between redox transitions, structural rearrangements, and the molecular basis of CPR’s interactions with its diverse redox partners.

## 2. Recent Advances in Techniques for Studying CPR ET Properties

Detailed characterization of the CPR-mediated ET cycle requires investigation of protein conformational dynamics while accounting for additional factors, including redox states, NADPH binding and dissociation, and interactions with redox partner proteins. To address these challenges, a variety of experimental methodologies have been applied over the past few decades. For instance, the combined use of biochemical measurements and X-ray crystallography has enabled researchers to correlate high-resolution structural information on CPR with its biological functions [22,25,36,37,38,39]. In addition, nanodisc technology has been employed to examine the orientation of CPR within a membrane-mimetic lipid bilayer, allowing assessment of its spatial configuration and the redox potentials of its flavin cofactors under physiologically relevant conditions [33,40,41]. Ultraviolet–visible (UV–Vis) spectroscopy has also been applied to distinguish the redox states of the FAD and FMN cofactors based on their characteristic absorption spectra, thereby facilitating the identification and monitoring of intermediate states [42,43,44]. While these approaches have yielded valuable quantitative insights into CPR function, recent efforts have increasingly shifted toward obtaining more detailed, physiologically relevant, and time-resolved mechanistic information. Current research aims to elucidate the dynamic conformational changes tightly coupled to CPR’s redox transitions and to capture transient intermediates and protein–partner interactions at higher resolution. These evolving objectives have driven the development and application of advanced biophysical and computational methodologies, which will be briefly discussed in the following sections.

The development of nonionic inulin-based polymer nanodiscs has further advanced the construction of CPR–P450 complexes in membrane-mimicking environments, even when the proteins carry opposite net charges. As mentioned above, the nanodisc technique enables the investigation of CPR orientations and ET properties in physiologically relevant membrane settings [33,40,41]. However, synthetic amphipathic polymers commonly used to form nanodiscs, such as poly(diisobutylene-*alt*-maleic acid) (DIBMA) or poly(styrene-*co*-maleic acid) (SMA), are enriched in charged and aromatic moieties that may engage in nonspecific interactions with membrane proteins, leading to destabilization and conformational changes. These properties limit the utility of traditional polymer nanodiscs for studying membrane-bound proteins with opposite net charges. The recently developed nonionic pentyl-functionalized inulin polymer (pentyl-inulin) addresses this limitation. Lacking ionic groups yet exhibiting strong nanodisc-forming ability when mixed with liposomes [45], pentyl-inulin enables the assembly of stable nanodiscs suitable for investigating the structure, dynamics, and functions of oppositely charged membrane proteins and protein complexes [46,47].

A particularly powerful strategy integrates site-specific incorporation of noncanonical amino acids (ncAAs) with single-molecule Förster resonance energy transfer (smFRET) [48] and site-directed spin labeling electron paramagnetic resonance (SDSL-EPR) [49,50]. These complementary techniques enable high-resolution distance measurements and provide dynamic structural insights across a broad range of conformational states and timescales. smFRET is especially effective for monitoring real-time domain motions within the 2–10 nm range, although its accuracy critically depends on the precise placement of fluorescent dyes, which are typically attached through cysteine residues. Because native cysteines in CPR are proposed to play functional roles, recent approaches have utilized orthogonal translation systems to incorporate ncAAs, such as *p*-propargyloxy-L-phenylalanine (PrF) and cyclopropene-L-lysine (CpK) [51]. These ncAAs provide unique bioorthogonal chemical handles, enabling efficient, site-specific, and minimally perturbative fluorescent dye conjugation. This strategy facilitates robust FRET efficiency measurements and accurate quantification of conformational changes under diverse biochemical conditions. In parallel, SDSL-EPR, when combined with the double electron–electron resonance (DEER) technique, yields distance distributions by detecting spin–spin dipolar interactions, which scale with a 1/r^3^ dependence [52,53,54]. Notably, ncAA-based spin labeling minimizes interference with protein function, and the paramagnetic FMN semiquinone state in CPR can, in principle, serve as a native spin center, allowing direct measurement of flavin-to-spin label distances. Together, these ncAA-enabled biophysical approaches constitute a versatile and highly effective toolkit for probing the structural and functional dynamics of CPR under diverse biochemical and redox conditions.

However, certain limitations should be recognized. Because CPR’s redox cofactors are strongly coupled to interdomain motions, signals from fluorescent or paramagnetic markers may be influenced by redox transitions, complicating interpretation. Furthermore, the spatial information obtained from these markers is restricted to local distance constraints between specific labeling sites, providing only limited coverage of the protein’s overall conformational landscape. As a result, they cannot fully characterize the conformational ensemble present in solution or detect subtle local rearrangements. These considerations indicate that while redox-sensitive markers are valuable tools, further refinement and integration with complementary approaches will greatly enhance their utility in studying CPR dynamics.

Beyond advanced biophysical techniques, computational approaches such as molecular dynamics (MD) simulations and quantum mechanics/molecular mechanics (QM/MM) methods have been increasingly applied to CPR systems. MD simulations, which typically rely on classical force fields, model the time-dependent motions of biomolecules at atomic detail [55]. They have been widely used to investigate processes ranging from ligand binding [56,57,58] to large-scale protein conformational changes [59,60,61,62,63,64], thereby providing mechanistic insights and revealing transient intermediate states that are often inaccessible to experimental techniques. Given the limited number of experimental structures of full-length CPR in distinct conformational states and in complex with its redox partner proteins, homology modeling and docking, in combination with MD simulations, continue to play a pivotal role in characterizing CPR conformational dynamics and protein–protein interactions.

Meanwhile, the QM/MM method, a multiscale computational approach that combines the electronic accuracy of quantum mechanics with the efficiency of classical molecular mechanics, has proven particularly powerful for probing chemical processes in complex biological environments [65]. Unlike purely quantum mechanical studies restricted to small model systems, QM/MM accounts for the influence of the surrounding protein environment on reactive processes. This approach has been extensively applied to diverse biological systems, including drug-metabolizing human P450s [66,67,68], human respiratory complex I [69,70], DNA/RNA polymerases [71,72], HIV-1 protease [73,74], and photosynthetic reaction centers [75,76]. When applied to CPR, QM/MM provides atomistic mechanistic insights that remain beyond the reach of experimental techniques alone.

## 3. Trends in Mechanistic Studies of the CPR-Mediated ET Cycle

A comprehensive understanding of the mechanisms underlying CPR-mediated ET processes, including the conformational dynamics of its domains, is essential given CPR’s pivotal role in supporting the catalytic cycles of its redox partner proteins, particularly P450s [7,8,9,10,11]. Investigating these mechanisms requires advanced methodologies capable of capturing both redox transitions and conformational rearrangements. In parallel, carefully designed experiments are needed to identify the key regulatory factors governing the CPR ET cycle. Over the past decade, a broad range of experimental and computational approaches have been applied to dissect mechanistic details at different stages of the cycle, including the initial NADPH-dependent reduction of FAD and FMN (priming reactions) [31], cofactor binding and release [21], and ET-coupled domain motions [25,26,27,28,29,77,78,79]. In the following sections, representative recent studies focusing on distinct mechanistic aspects of the CPR ET cycle will be summarized to highlight these advances and outline potential future directions.

### 3.1. NADPH Binding and NADP^+^ Dissociation

**Figure 3:**
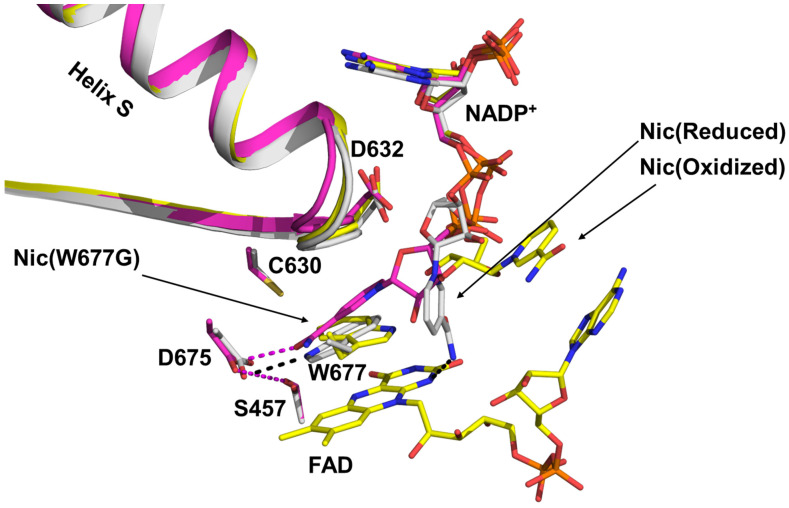
Superimposed structures illustrating the transition of the NADPH ribityl–nicotinamide tail from the unbound state (yellow) to the final stacked state, positioned for hydride transfer to the FAD isoalloxazine ring (magenta) [21]. The “Nic” label in the figure denotes the nicotinamide tail of NADPH.

The binding and dissociation of NADPH/NADP^+^ have long been central topics in studies of the CPR-mediated ET cycle, owing to NADPH’s intrinsic role as an electron donor and its potential influence on CPR conformational dynamics [21,24,77,78,80,81,82]. Using structural and kinetic analyses of both wild-type and mutant rat CPR (rCPR), Kim and coworkers characterized the structural and mechanistic basis of NADPH binding [21,83]. By comparing crystallographic structures of rCPR at different stages of NADPH engagement, they proposed a stepwise mechanism. Binding begins with the interaction of the 2′-AMP-PP_i_ moiety of NADPH with Arg597 and Lys602 of rCPR. The pyrophosphate group subsequently interacts with Arg567 and Arg298, stabilizing the 2′-AMP-5′PP_i_ segment of NADPH. This is followed by a conformational transition of the Asp632 loop from an extended to a retracted state, which induces rotation of the indole ring of Trp677. This arrangement positions the ribityl–nicotinamide tail of NADPH to stack against the *re* face of the FAD isoalloxazine ring, thereby initiating hydride transfer to FAD (Figure 3).

**Figure 4:**
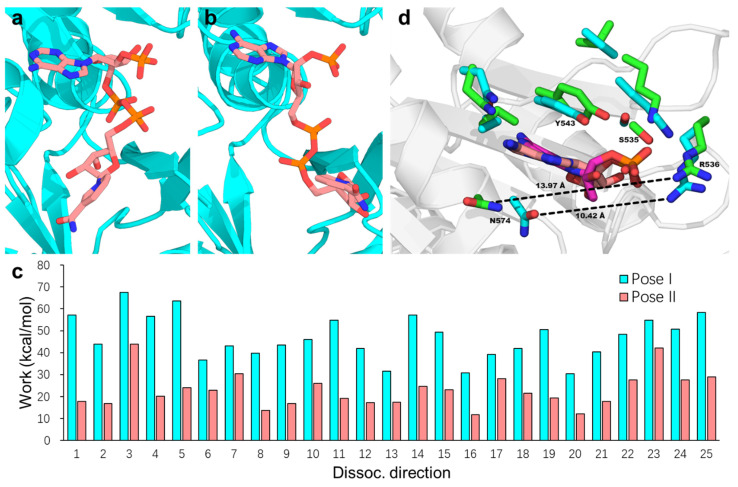
Representative of NADP^+^ poses I (**a**) and II (**b**) within binding pocket during the MD simulation. (**c**) Work calculated from the biasing force to extract NADP^+^ from the binding pocket and transfer it into the bulk solvent along the 25 dissociation pathways, shown for pose I (cyan) and pose II (salmon). (**d**) Local binding environment of the NADP^+^ adenosine moiety in poses I and II, obtained by superimposing the FAD-binding domain structures. For clarity, only the adenosine groups in the two binding poses are shown, with carbon atoms in poses I and II colored salmon and magenta, respectively. Residues surrounding the adenosine group are shown in licorice representation, with side-chain carbon atoms colored cyan (pose I) and green (pose II). The binding pocket gate width in these poses is indicated by the distance between the amide C atom of N574 and the amine N atom of R536, shown as black dashed lines and measured in Å. Reproduced with permission from Ref. [84]. Copyright 2024 American Chemical Society.

Once hydride transfer from NADPH to FAD is complete, the side chain of Trp677 displaces the positively charged nicotinamide tail of the resulting NADP^+^, initiating its dissociation. Building on the hypothesis that NADP^+^ dissociation follows the same pathway as NADPH binding [21], Xia and Hirao recently employed microsecond-scale MD simulations combined with free-energy calculations to investigate the dissociation pathways of NADP^+^ from rCPR [84]. Their results indicate that the adenine head of NADP^+^ undergoes a conformational rearrangement prior to exiting the binding pocket. This process is facilitated by rotation of the O–C–N–C dihedral angle from the most stable pose (pose I, Figure 4a) to an alternative pose (pose II, Figure 4b), which is 1.53 kcal/mol less stable than pose I. Further analysis revealed that the average work calculated from the biasing force to displace NADP^+^ from the rCPR binding pocket was significantly lower for pose II (22.89 kcal/mol) than for pose I (47.13 kcal/mol) (Figure 4c), suggesting that the pose II pathway imposes a reduced energetic barrier to dissociation. Examination of the local binding environment of the NADP^+^ adenine moiety showed that the transition from pose I to pose II disrupts key intermolecular interactions between NADP^+^ and the protein. Additionally, this conformational change results in the opening of the binding-pocket gate formed by Arg536 and Asn574 (corresponding to Arg597 and Asn635 in Refs. [21,83]) by approximately 3.55 Å, which constitutes a major factor facilitating NADP^+^ dissociation (Figure 4d).

### 3.2. Electron Transfer Processes Within CPR

Elucidating the mechanisms of ET between NADPH, FAD, and FMN, as well as identifying the key residues involved, is essential for understanding the catalytic cycle of CPR. Before entering the canonical CPR-mediated ET cycle, which begins from the one-electron reduced FAD–FMNH^•^ state, the protein undergoes a priming reaction that involves the formation of the disemiquinone species FADH^•^ and FMNH^•^ [20]. A recent computational study by Li et al. investigated this priming step to characterize the ET pathway from the initial electron donor NADPH to the intermediate acceptor FAD and subsequently to the FMN cofactor [31]. Upon NADPH binding, a hydride equivalent (two electrons and a proton) is transferred from the nicotinamide moiety of NADPH to the isoalloxazine ring of FAD, accompanied by proton transfer, with an energy barrier of 12.9 kcal/mol. The resulting fully reduced FADH^−^ then sequentially transfers electrons to the adjacent FMN molecule: the first interflavin ET forms the disemiquinone pair (FADH^•^ and FMNH^•^), and the second generates oxidized FAD and fully reduced FMNH^−^. Both interflavin ET steps proceed via a proton-coupled electron transfer (PCET) mechanism. In the first PCET, ET from FADH^−^ to FMN is accompanied by proton donation from Glu142 to FMN through a water-mediated pathway involving two water molecules. In the second PCET, ET is coupled with proton shuttling from the FADH^•^ semiquinone to Asp675, facilitated by Ser457, consistent with mutagenesis studies showing that CPR activity is reduced in the Asp675Asn/Ser457Ala mutant [85]. Furthermore, Li et al. examined how the protonation state of the His180 imidazole ring influences the first interflavin PCET. QM/MM calculations showed that when His180 is in the doubly protonated cationic form (rather than the δ-protonated neutral form), the energy barrier for ET from FADH^−^ to FMN is lowered by 7.5 kcal/mol, and the reaction product is stabilized by 11.2 kcal/mol.

### 3.3. Factors Relevant to the CPR Conformational Change

To facilitate both interflavin ET from FAD to FMN and ET from FMN to its redox partner protein, CPR must undergo a substantial conformational change from a compact closed state, where the distance between the two cofactors is ~4 Å, to an extended open state, where this distance exceeds 60 Å [1]. Extensive efforts have been made to elucidate the mechanisms underlying this closed-to-open transition, and several contributing factors have been proposed, including the flexibility of the hinge segment [25,86], the influence of ionic strength [53,77,87], the binding and release of NADPH/NADP^+^ [21,24,77,78,80,81,82], and interactions with redox partner proteins [88].

**Figure 5:**
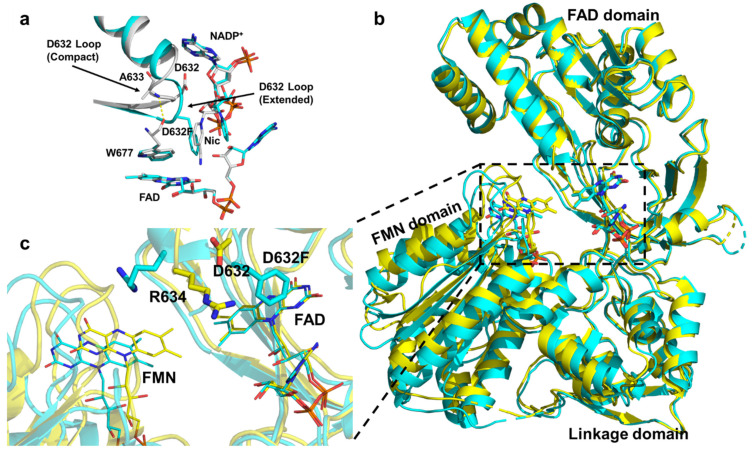
(**a**) The Asp632 loop undergoes an extended-to-compact conformational change to accommodate binding of the NADPH ribityl–nicotinamide tail. (**b**) Structural comparison showing a ~2 Å greater separation between the FAD and FMN domains in the Asp632Phe mutant relative to the wild type. Superimposed structures of the oxidized wild-type CPR (gold) and oxidized Asp632Phe mutant (cyan). (**c**) The lower-left panel provides an enlarged view of the dashed rectangular area in the right panel. In the Asp632Phe mutant, the FMN is displaced by ~2 Å farther from FAD compared with the oxidized wild type [21].

A recent experimental study by Xia et al. suggested that the coordinated movements of Trp677 and the Asp632 loop play a central role in NADPH binding, NADP^+^ release, and initiation of the closed-to-open transition of CPR [21]. By integrating site-directed mutagenesis with X-ray crystallography, they showed that during NADPH binding, Trp677 reorients its indole ring to accommodate the nicotinamide moiety of NADPH. This rotation induces an extended-to-compact conformational change of the Asp632 loop through the formation of a hydrogen bond between the backbone N–H of Trp677 and the carbonyl of Ala633 (Figure 5a). The resulting loop retraction promotes a more compact CPR conformation, thereby facilitating interflavin ET. These coupled motions align with previous observations showing that NADPH binding stabilizes the compact state and accelerates interflavin ET [28,77,89]. Following FAD reduction, the indole ring of Trp677 rotates back to displace the positively charged NADP^+^ tail, triggering extension of the Asp632 loop. Structural comparisons between wild-type rCPR (where the Asp632 loop is compact) and the Asp632Phe mutant (where the loop remains extended) revealed that Arg634 in the mutant adopts an alternative orientation, with its side chain pointing toward the FMN domain, causing the FMN cofactor to shift by ~2 Å away from FAD relative to the wild type. Xia et al. thus proposed that the loop extension drives Arg634 side-chain rotation toward the FMN domain, thereby facilitating CPR opening (Figure 5b). However, this model does not appear fully consistent with the kinetic behavior of the Arg634Ala mutant, which exhibits increased activity toward CYP2B4 and cytochrome *c*.

A more recent study by Bizet and co-workers employed SDSL-EPR spectroscopy combined with the DEER technique to investigate the conformational dynamics of human CPR (hCPR) [53]. Nitroxide spin labels were introduced at two specific residues, Gln157 and Lys668, located in the FMN and FAD domains, respectively. This labeling enabled monitoring of structural dynamics in the one-electron-reduced enzyme by measuring distances between the semiquinone form of the FMNH^•^ cofactor and the labeled residues. Analysis under varying NaCl concentrations revealed that the FMNH^•^–Gln157 distance remained largely unchanged, indicating that ionic strength does not significantly alter the binding orientation of the FMN cofactor. In contrast, DEER measurements showed that at high NaCl concentration (50 mM), the FMNH^•^–Lys668 distance distribution shifted from ~30 Å to ~50 Å, suggesting that increased ionic strength promotes a shift in CPR’s conformational equilibrium from the closed to the open state in solution. Complementary MD simulations initiated from both closed and open conformations under different salt concentrations supported these findings. Simulations starting from the closed state maintained short interflavin distances under all salt conditions, whereas those starting from the open state rapidly transitioned to the closed conformation at low salt but remained open at high salt concentrations. Notably, ionic-strength-dependent modulation of CPR’s conformational equilibrium has also been observed in other biophysical studies [51,90].

### 3.4. Protein–Protein Interactions and Electron Transfer Pathways Between CPR and Its Redox Partners

For the CPR-mediated ET cycle to deliver electrons to an acceptor (typically a heme), CPR must adopt an open conformation that enables complex formation with its redox partner. On the ER membrane, CPR is expressed at considerably lower levels than its redox partners [32,91], increasing the likelihood that each CPR molecule transfers electrons to multiple structurally diverse proteins. These partners vary in sequence length (typically 100–400 amino acids) and exhibit substantial differences in their binding interfaces. Consequently, elucidating the relative orientation of CPR and its partners within such complexes, identifying residues critical for the protein–protein interface, and defining the potential ET pathways from the FMN cofactor to the electron acceptor are central to understanding CPR-mediated ET.

To address this knowledge gap, Sugishima and co-workers solved the crystal structure of a hinge-shortened rCPR variant (ΔTGEE) in complex with one of its redox partners, rat heme oxygenase-1 (rHO-1) [39]. Kinetic analyses showed that the ΔTGEE variant retained the ability to transfer electrons to rHO-1, albeit with markedly reduced efficiency. The crystal structure of the ΔTGEE–rHO-1 complex revealed that the FMN domain of rCPR interacts with Lys149 and Lys153 of rHO-1, whereas the FAD domain contacts Arg185. The structural data also suggested that binding of NADPH or NADP^+^ shifts rCPR’s conformational equilibrium toward a partially open state, thereby exposing the FMN cofactor at the protein surface and facilitating complex formation with its redox partner.

**Figure 6:**
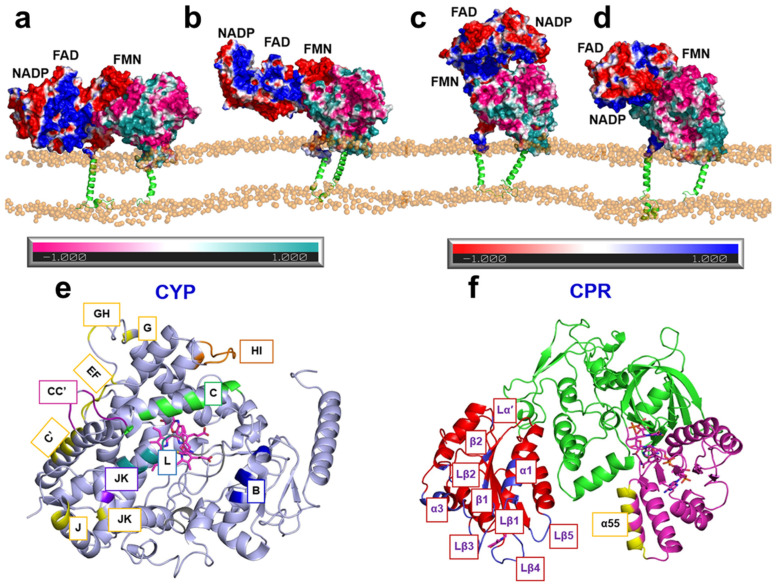
(**a**–**d**) Representative structures of the hCPR–hCYP1A1 complex from MD simulation trajectories. Soluble domains are shown as surfaces and transmembrane helices as cartoons. Electrostatic potentials are mapped onto the surfaces: negatively charged regions of hCPR and hCYP1A1 are colored red and pink, respectively, while positively charged regions are colored blue and cyan, respectively. (**e**,**f**) 3D structures of hCYP1A1 (**e**) and hCPR (**f**) shown as cartoon models, with interaction regions highlighted in different colors [93]. Adapted, under a CC-BY-NC-ND 4.0 license, from [93].

Despite significant advances from structural studies, the orientation of CPR in complex with its primary redox partners, the P450 enzymes, remains incompletely understood. Recently, MD simulations have been applied to investigate the conformational dynamics of CPR in complexes with various P450 isoforms [23,84,92,93,94,95]. To identify plausible CPR–CYP1A1 complex structures, Mukherjee et al. first applied Brownian dynamics docking to generate six potential encounter complexes between hCPR and human CYP1A1 (hCYP1A1) [93]. All six were stabilized by complementary electrostatic interactions between the negatively charged surface of the hCPR FMN domain and the positively charged surface of hCYP1A1. These docked complexes were then refined through “soluble” MD simulations. Of these, three underwent structural reorientations that disrupted their ET capability or altered the FMN binding pose and were therefore excluded from further analysis. The remaining hCPR–hCYP1A1 complexes were embedded in a phospholipid bilayer to examine their orientations in a membrane environment. The simulations revealed notable reorientations of both hCPR and hCYP1A1 in the presence of the membrane (Figure 6a–d). Specifically, the globular domain of hCYP1A1 became less deeply inserted into the membrane, likely increasing the interfacial area available for interaction with hCPR. In parallel, the hCPR FMN domain shifted closer to the phospholipid layer, facilitated by the flexible loop connecting the FMN domain to the transmembrane (TM) helix, a region previously proposed to play a critical role in CPR function [96,97]. The FAD domain was also observed to reorient toward the proximal surface of hCYP1A1, resulting in an interflavin distance of ~44.7 Å. This distance falls between those previously reported for the closed (14.2 Å) and fully open (61.3 Å) conformations determined by X-ray crystallography [37] and is somewhat larger than that observed in the rCPR–rHO-1 complex (22 Å [39]).

Furthermore, Mukherjee et al. performed a detailed analysis of the MD trajectories and identified a network of specific interfacial contacts between hCPR and hCYP1A1 that stabilize the complex and may facilitate efficient ET. Several key residues in hCPR, located on the α1 and α3 helices, the Lα′ and Lβ1-5 loops, and the β1 and β2 strands of the FMN domain, as well as on helices B, C, and L and loops J and K of hCYP1A1 (Figure 6e,f), were found to form extensive interactions that maintained complex stability during the simulations. Among these, Glu142, Asp144, and Glu179 in the FMN domain, together with Lys668 in the FAD domain, have also been implicated in other CPR–P450 interaction studies [23,92,94,98,99]. In addition, two plausible ET pathways from the hCPR FMN cofactor to the heme iron center of hCYP1A1 were identified: the first proceeds via FMN–Ile458–Cys457–heme, while the second follows FMN–Lys456–Cys457–heme. The involvement of Ile458 and Lys456 in hCYP1A1, or residues at equivalent positions in other enzymes, has been supported by multiple experimental and computational studies on various CPR–P450 systems [23,100,101,102,103], while Cys457 in CYP1A1 serves as the heme-coordinating cysteine. Collectively, these findings advance our understanding of CPR orientation in complexes with its redox partners and provide important mechanistic insights into the CPR-mediated ET cycle.

## 4. Conclusions and Outlook

In this Perspective, we have reviewed recent advances in experimental and computational approaches used to investigate the mechanisms underlying the CPR-mediated ET cycle, highlighting selected case studies. These studies have clarified several previously unresolved aspects of CPR function, particularly regarding its complex formation with redox partners and the associated ET processes. Nevertheless, the intrinsic complexity of CPR’s structure and its ET cycle continues to pose obstacles to a comprehensive understanding of how CPR’s redox states coordinate with structural rearrangements during ET to different partners.

One major challenge lies in identifying the factors that control or gate the equilibrium between CPR conformational states. A domain-opening mechanism has been proposed involving sequential sub-events, beginning with FAD reduction, followed by interflavin ET, Trp677 indole ring rearrangement, NADP^+^ dissociation, Asp632 loop extension, and ultimately CPR opening [21]. However, the precise order of these events remains uncertain. Addressing this issue will require carefully designed experiments capable of independently monitoring each step with sufficient temporal resolution to resolve their sequence. Computational approaches such as MD simulations also hold considerable promise for probing how protein dynamics and local structural rearrangements influence CPR function. Although not yet widely applied to this specific question, such methods have been successfully employed in studies of other biological systems [62,63,64] and in analyses of CPR–P450 complex dynamics [23,92,93,94].

A comprehensive understanding of CPR orientation within the membrane and its spatial relationship to redox partner proteins remains another critical goal for elucidating the molecular mechanisms of membrane-associated ET. The membrane environment strongly influences the structure and function of membrane proteins [104,105,106]. Moreover, CPR’s numerous redox partners differ substantially in structure and ET requirements, making it essential to define the specific interactions of CPR with each partner. The intrinsic flexibility of CPR’s hinge segment enables large-scale domain rearrangements that modulate binding modes, ET pathways, and catalytic efficiency. Understanding how these dynamic motions coordinate with partner-specific recognition will advance mechanistic insight and guide the rational engineering of CPR–partner systems for biomedical and biotechnological applications.

Despite the biological importance of these interactions, all currently available CPR structures are truncated and lack the N-terminal transmembrane helix, while structural data on intact CPR–redox partner complexes remain scarce. These gaps limit our understanding of CPR in its native membrane-bound state and of the structural determinants governing CPR–partner interactions. Recent advances in cryo-electron microscopy (cryo-EM) and artificial intelligence (AI)-based structural prediction may help overcome these challenges. Cryo-EM, aided by optimized detergents, now enables the study of membrane proteins without the need for well-diffracting crystals [107,108,109]. AI-based tools such as AlphaFold [110] have demonstrated remarkable capabilities in predicting structures of previously uncharacterized proteins and protein–protein complexes. By leveraging evolutionary and structural information, these models can provide highly accurate predictions even in the absence of close homologs, offering valuable insights into protein function and molecular interactions.

In the coming decades, continued progress in both experimental and computational methodologies is expected to further advance our understanding of the structural and mechanistic complexities of CPR and its ET cycle. The integration of high-resolution experimental techniques with robust theoretical frameworks, combined with expanding computational power, will likely enable major breakthroughs in elucidating the molecular details of CPR–redox partner interactions and in uncovering how ET processes are regulated.

## Data Availability

Not applicable.
